# The Expression of Proteins Related to Serotonin Pathway in Pulmonary Arteries of Dogs Affected With Pulmonary Hypertension Secondary to Degenerative Mitral Valve Disease

**DOI:** 10.3389/fvets.2020.612130

**Published:** 2020-12-03

**Authors:** Siriwan Sakarin, Sirilak Disatian Surachetpong, Anudep Rungsipipat

**Affiliations:** ^1^Department of Veterinary Medicine, Faculty of Veterinary Science, Chulalongkorn University, Bangkok, Thailand; ^2^Companion Animal Cancer Research Unit, Department of Veterinary Pathology, Faculty of Veterinary Science, Chulalongkorn University, Bangkok, Thailand

**Keywords:** degenerative mitral valve disease, hyperplasia, pulmonary artery, pulmonary hypertension, serotonin, smooth muscle cells

## Abstract

**Background:** Pulmonary hypertension (PH) can cause medial thickening, a hallmark of pulmonary arterial remodeling. The serotonin (5HT) pathway has been suggested as a factor associated with PH by inducing pulmonary arterial smooth muscle cells (SMCs) proliferation, a major cause of medial thickening. This study aims to demonstrate the expression of molecules in the 5HT pathway in the pulmonary artery of dogs affected with PH secondary to degenerative mitral valve disease (DMVD) compared to DMVD and healthy control dogs.

**Materials and Methods:** The study included lung samples from the carcasses of 19 older small-breed dogs (Control *n* = 5, DMVD *n* = 7, DMVD+PH *n* = 7). Lung tissue sections were performed Hematoxylin and Eosin staining for measuring the percentage of medial thickness and immunohistochemistry for evaluating the expression of proteins in the 5HT pathway including serotonin transporter (SERT), serotonin 2A receptor (5HT2A), tryptophan hydroxylase 1 (TPH1), extracellular regulated kinase 1/2 (ERK1/2), and phosphorylated ERK1/2 (pERK1/2).

**Results:** Medial thickening of the pulmonary arteries was found in the DMVD and DMVD+PH groups compared to the control. The medial thickening of the DMVD+PH group was increased significantly compared to that in the DMVD group. Intracytoplasmic expression of proteins related to the 5HT pathway was mainly presented in the medial layer of the pulmonary arteries. The control group showed a low expression of proteins related to the 5HT pathway. An intensive expression of SERT, 5HT2A, TPH1, and ERK1/2 protein was seen in the DMVD and DMVD+PH groups. Interestingly, pERK1/2 was strongly represented only in the DMVD+PH group.

**Conclusions:** Overexpression of proteins related to the 5HT pathway including SERT, 5HT2A, TPH1, ERK1/2, and pERK1/2 was associated with medial remodeling in dogs affected with secondary to DMVD.

## Introduction

Pulmonary hypertension (PH) has been defined as an abnormal increase in pulmonary arterial pressure (PAP). Several causes can mediate PH in dogs. Among these causes, left heart disease is suggested as being the most common cause of PH in dogs (1–4). Degenerative mitral valve disease (DMVD) is a common left heart disease in older small-sized breed dogs. It is a progressive disease of valve degeneration that impacts cardiovascular hemodynamics. Congestive heart failure and PH are common complications of late-stage DMVD (2, 5, 6).

The clinical presentation including syncope, respiratory distress and exercise intolerance are signs suggestive of PH. Right heart catheterization is a gold standard method for measuring PAP in humans, whereas echocardiography is an acceptable method for estimating PAP in veterinary medicine. According to the ACVIM consensus guideline for the diagnosis and treatment of PH in dogs, estimated PAP and anatomical cardiac changes assessed by echocardiography have been used to evaluate the probability of PH in dogs (4).

Pulmonary arterial remodeling, especially medial thickening is a hallmark of structural changes of the pulmonary artery presenting in all forms of PH in human patients (7–12), PH-induced animal models (13–17) as well as dogs affected with PH secondary to DMVD (3). However, the etiology of this pathological change remains unclear.

Serotonin pathway has been suggested as being associated with PH by inducing pulmonary arterial smooth muscle cells (SMCs) proliferation, which is a major cause of medial thickening (18). Serotonin or 5-hydroxytryptamine (5HT) is synthesized from tryptophan through tryptophan hydroxylase 1 (TPH1), a rate-limiting enzyme in pulmonary arterial endothelial cells. Serotonin then stimulates pulmonary arterial SMCs proliferation in a paracrine fashion causing medial thickening. These effects of 5HT are mediated via the 5HT transporter (SERT) and 5HT receptors (19, 20). Although mechanisms of 5HT mediated pulmonary arterial SMCs proliferation remain unclear, several studies suggest 5HT transport into pulmonary arterial SMCs via SERT and 5HT receptors. Intracellular accumulation of 5HT induces reactive oxygen species and Rho-kinase activating phosphorylation and nuclear translocation of extracellular regulated kinase 1/2 (ERK1/2), leading to an increase in transcription of nuclear growth factors and mediating cellular proliferation (21, 22). The serotonin pathway has been of interest as a factor associated with PH due to evidence showing that patients who use drugs which are SERT substrates develop PH (19, 20, 23). In addition, a study in PH-induced animal models demonstrated that inhibition of SERT and 5HT receptors could reduce the risk of PH development (24).

Overexpression of proteins in the 5HT pathway has been suggested as being involved in PH by stimulating pulmonary artery SMCs proliferation (22). Previous studies reported an overexpression of SERT in human patients affected with primary PH (18, 25). A study in PH-induced animal models showed that not only SERT but also 5HT receptor overexpression was associated with pulmonary arterial SMCs hyperplasia (26). Although there is evidence supporting that the 5HT pathway is associated with the development of PH in both human patients and animal models, its effects on the pathogenesis of PH in DMVD dogs were not evaluated. This study aimed to demonstrate the expression of proteins related to the 5HT pathway in the pulmonary artery of dogs affected with PH secondary to DMVD compared to DMVD and healthy control dogs.

## Materials and Methods

### Animals

**Figure 1:**
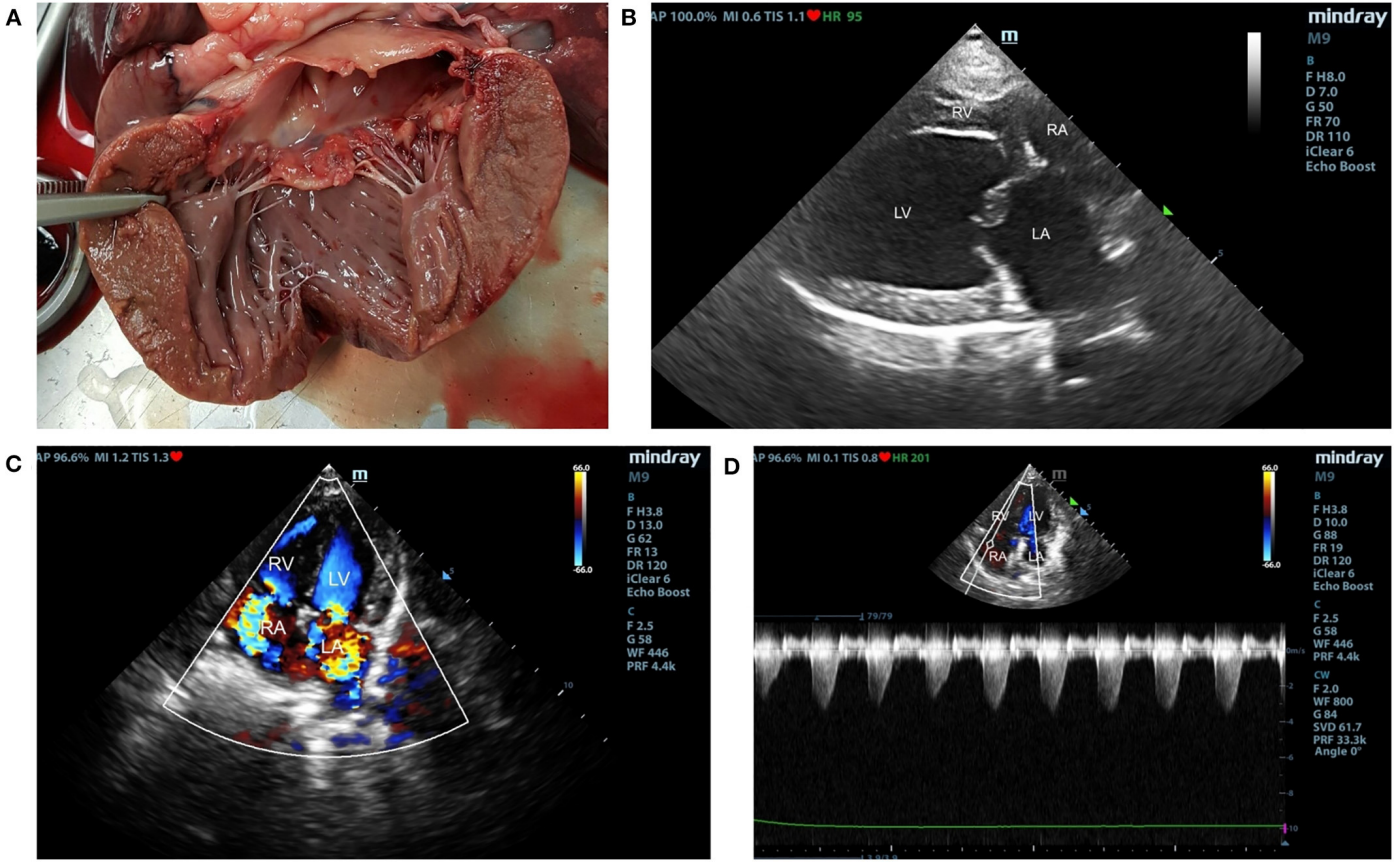
The gross pathology of mitral valve leaflet **(A)** and echocardiographic findings of degenerative mitral valve disease (DMVD) dogs with pulmonary hypertension (PH) **(B–D)**. The affected mitral valve leaflets are nodular and thickening **(A)**. Echocardiography from two-dimensional right parasternal four chamber view showed thickening of mitral valve leaflets and left atrial (LA) enlargement **(B)**. Color-flow Doppler echocardiography shows mitral valve and tricuspid valve regurgitation jets **(C)**. Spectral Doppler echocardiography of tricuspid regurgitation in DMVD dogs with PH. Peak systolic tricuspid regurgitation velocity were measured to evaluate estimated pulmonary arterial pressure (PAP) **(D)**.

Carcasses of 19 elder small-breed dogs presented at the Department of Pathology, Faculty of Veterinary Science, Chulalongkorn University for necropsy were selected into the study. The Ethical approval is not required because the study was performed in donation cadavers. Dogs were divided into three groups, including control (*n* = 5), degenerative mitral valve disease (DMVD) (*n* = 7) and degenerative mitral valve disease with pulmonary hypertension (DMVD+PH) groups (*n* = 7), based on mitral valve thickness measured at necropsy and previous clinical diagnostic records. Dogs were excluded, if they had pulmonary diseases, heartworm infection, systemic hypertension, neoplasia, and other systemic diseases such as hepatic and kidney diseases evaluated by antemortem diagnostic history and gross pathology. Dogs with mitral valve thickness <2 mm measured by Vernier caliper were included in the control group (27, 28). Dogs affected with DMVD had to have mitral valve thickness >2 mm and had been diagnosed with DMVD stage C before death. All DMVD dogs had to have vertebral heart score more than 10.5 with either current or past signs of pulmonary edema assessed by radiography, left atrial and ventricular enlargement [LA/Ao ≥ 1.6 (Swedish method) and left ventricular internal diameter during diastole normalized with the Allometric scale method (LVIDdN) ≥ 1.7] (6). Dogs in the DMVD+PH group had to have an intermediate to high probability of PH secondary to DMVD evaluated by estimated PAP and anatomic structural changes assessed by echocardiography (Figure 1) (4, 29).

Lung tissues were collected at peripheral regions of right caudal lung lobes and preserved in 10% neutral buffered formalin for 24 h and embedded in paraffin blocks. Lung tissue sections (4 μm thickness) were immunohistologically stained for proteins related to 5HT pathway including SERT, 5HT2A, ERK1/2, pERK1/2, and TPH1 following the modified protocols described by previous studies (15, 16, 18, 25, 28, 30–32). Briefly, tissue sections were deparaffinized, pre-treated with citrate buffer, pH 6.0, blocked endogenous peroxidase activity and non-specific antibody binding. The sections were incubated with primary antibodies including monoclonal mouse anti-SERT antibody (Advanced Targeting Systems, Cat# AB-N40, RRID:AB_2868504), monoclonal mouse anti-5HT2A (Santa Cruz Biotechnology, Cat# sc-166775, RRID:AB_2233203), monoclonal rabbit anti-ERK1/2 and pERK1/2 (Cell Signaling Technology, Cat# 8201, RRID:AB_10695902), and monoclonal mouse anti-TPH1 (Sigma-Aldrich, Cat# T0678, RRID:AB_261587) at dilution 1:200 overnight. Tissue sections were then incubated with horse radish peroxidase-labeled polymer conjugated with secondary antibody (Dako, Cat# K5007). Sections were incubated with 3,3′-diaminobenzidine tetrahydrochloride (Dako, Cat# K5007) diluted 1:50 for visualizing peroxidase activity and counterstained with Mayer's hematoxylin.

Ten pulmonary arteries with the external diameter approximately 300 μm from each dog were microscopically examined under a light microscope and randomly photographed with a photomicroscope (Olympus BX50®, Olympus Optical, Tokyo, Japan) at 100x magnification for measuring internal and external diameters and calculating the percentage of medial thickness (%MT) as following equation: (external diameter – internal diameter)/external diameter × 100. Five areas of each pulmonary artery were photographed at 400x magnification for measuring the positive areas using an image analyzer program (Image-Pro® Plus Software, RRID:SCR_007369, version #6.0). The percentage of the positive area was calculated by dividing the positive area by the total area. The average positive areas of each antibody were calculated.

### Statistical Analysis

Statistical analysis was performed by the computer-based software (SPSS, RRID:SCR_002865). The Shapiro-Wilk test was used for the normality test. Normally distributed data were presented as mean ± standard deviation (SD). One-way ANOVA was used for evaluating differences among the control, DMVD and DMVD+PH groups. LSD test was used for *post-hoc* analysis and the *p*-value for pairwise comparison was adjusted by Bonferroni correction *p*-value of <0.05 was considered statistically significant.

## Results

### Clinical Characteristics of Dogs

The characteristics of dogs recruited to the study are summarized in Table 1. Age, weight, and sex of dogs in the control, DMVD and DMVD+PH groups were not different. The thickness of mitral valve in the DMVD and DMVD+PH groups was significantly thicker than the control group (Table 1). The cause of death of DMVD dogs with and without PH was cardiovascular failure. Only one dog in the DMVD+PH group was euthanized due to non-response to cardiovascular drugs. Dogs in the control group died secondary to postoperative complications and the car accident. None of dogs in the control group had abnormality of heart and lungs. Based on antemortem echocardiographic findings, LA/Ao and LVIDdN was not different between the DMVD and DMVD+PH groups (Table 1). The average estimated PAP of dogs in the DMVD+PH groups was 75.77 ± 28.79 mmHg (range 49.51–116 mmHg). Five of seven dogs in the DMVD+PH group had anatomic changes including right side of the heart and pulmonary artery enlargement.

**Table 1:** The clinical characteristic of dog in the control, degenerative mitral valve disease (DMVD), and degenerative mitral valve disease with pulmonary hypertension (DMVD+PH) groups.

**Parameters**	**Control (*n* = 5)**	**DMVD (*n* = 7)**	**DMVD+PH (*n* = 7)**	***p*-value**
Age (year)	11.20 ± 3.63	13.71 ± 1.80	13.86 ± 1.57	0.136
Weight (kg)	5.59 ± 0.38	4.91 ± 1.49	6.25 ± 2.99	0.486
Sex				
Male	3 (M = 1; Mc = 2)	3 (M = 1; Mc = 2)	4 (M = 2; Mc = 2)	
Female	2 (F = 1; Fs = 1)	4 (F = 2; Fs = 2)	3 (F = 2; Fs = 1)	
Breed	Shih Tzu (*n* = 3), Mixed (*n* = 1), Pomeranian (*n* = 1)	Poodle (*n* = 4), Shih Tzu (*n* = 2), Pomeranian (*n* = 1)	Poodle (*n* = 2), Chihuahua (*n* = 2), Pomeranian (*n* = 1), Schnauzer (*n* = 1), Shih Tzu (*n* = 1)	
Mitral valve thickness (mm)	0.60 ± 0.21	2.40 ± 0.16^a^	2.54 ± 0.15^a^	^a^ <0.0001
LA/Ao	-	2.05 ± 0.36	2.24 ± 0.79	0.595
LVIDdN	-	2.08 ± 0.41	1.71 ± 0.39	0.142

*Data are expressed as mean ± standard deviation (SD)*.

*The significant difference was assessed by the one-way ANOVA at p < 0.05*.

a *Indicate significant difference at p < 0.05 compared to the control group*.

### Percentage of Medial Thickness

**Figure 2:**
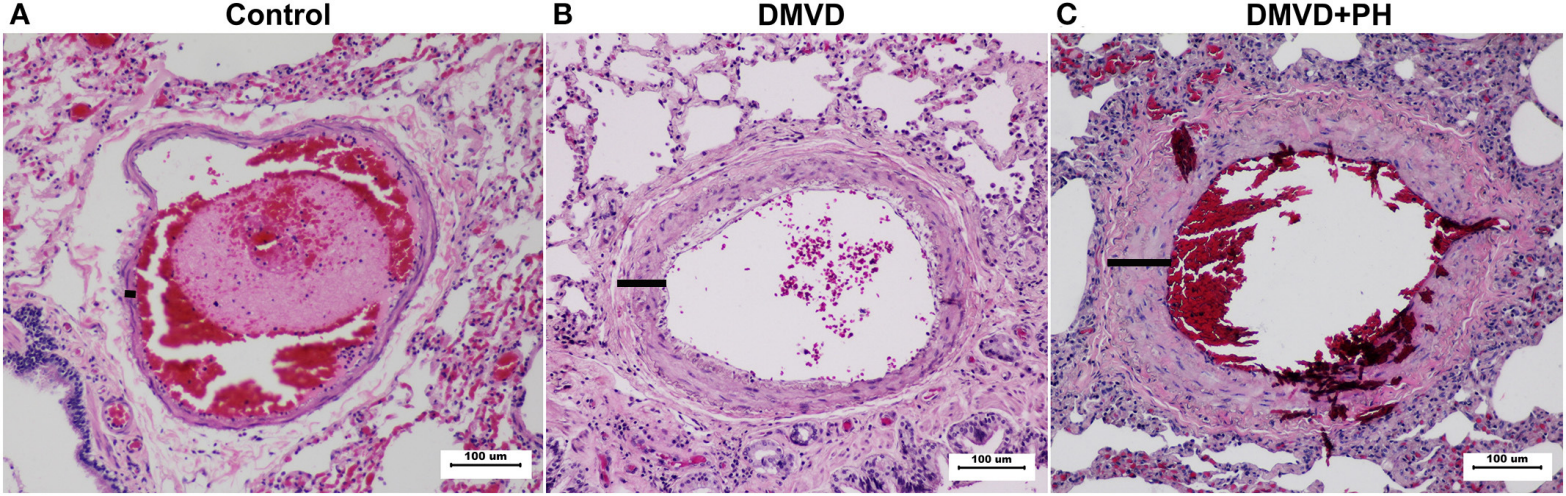
The histopathology of the pulmonary artery in the control **(A)**, degenerative mitral valve disease (DMVD) **(B)** and degenerative mitral valve disease with pulmonary hypertension (DMVD+PH) **(C)** groups (Hematoxylin and eosin stain, 100x magnification). Medial thickness of the pulmonary arteries were increased in the DMVD and DMVD+PH groups compared to the control group.

Pulmonary arteries in dogs with DMVD and DMVD with PH were presented with medial thickening (Figure 2). The percentage of medial thickness (%MT) was increased in the DMVD (22.36 ± 1.44%) and DMVD+PH groups (32.25 ± 5.06%) compared to the control group (11.04 ± 0.95%) (*p* < 0.0001). In addition, %MT was increased in the DMVD+PH group compared to that in the DMVD group (*p* < 0.0001). An increase in smooth muscle layers was seen in the DMVD and DMVD+PH groups.

### Immunolocalization of Proteins Related to in 5HT Pathway

**Figure 3:**
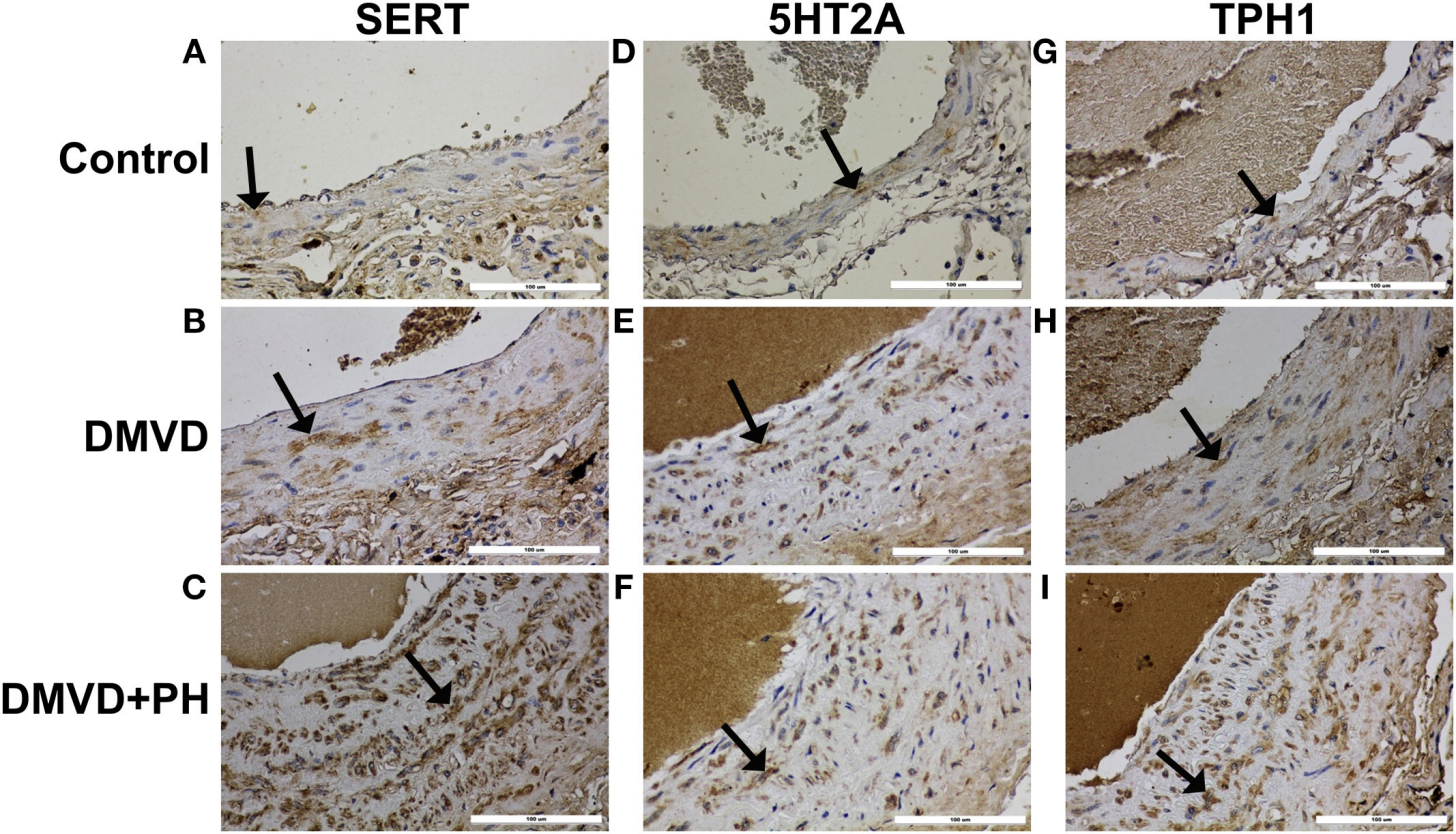
The expression of protein related to 5HT pathway including serotonin transporter (SERT), serotonin 2A receptor (5HT2A), tryptophan hydroxylase 1 (TPH1) in pulmonary arterial smooth muscle cells of the control **(A,D,G)**, degenerative mitral valve disease (DMVD) **(B,E,H)** and degenerative mitral valve disease with pulmonary hypertension (DMVD+PH) groups **(C,F,I)**. The SERT, 5HT2A and TPH1 expression in cytoplasm of pulmonary arterial smooth muscle cells presented as brown color (Labeled streptavidin-biotin, Immunohistochemistry, Mayer's Hematoxylin counterstained, 400x magnification).

**Figure 4:**
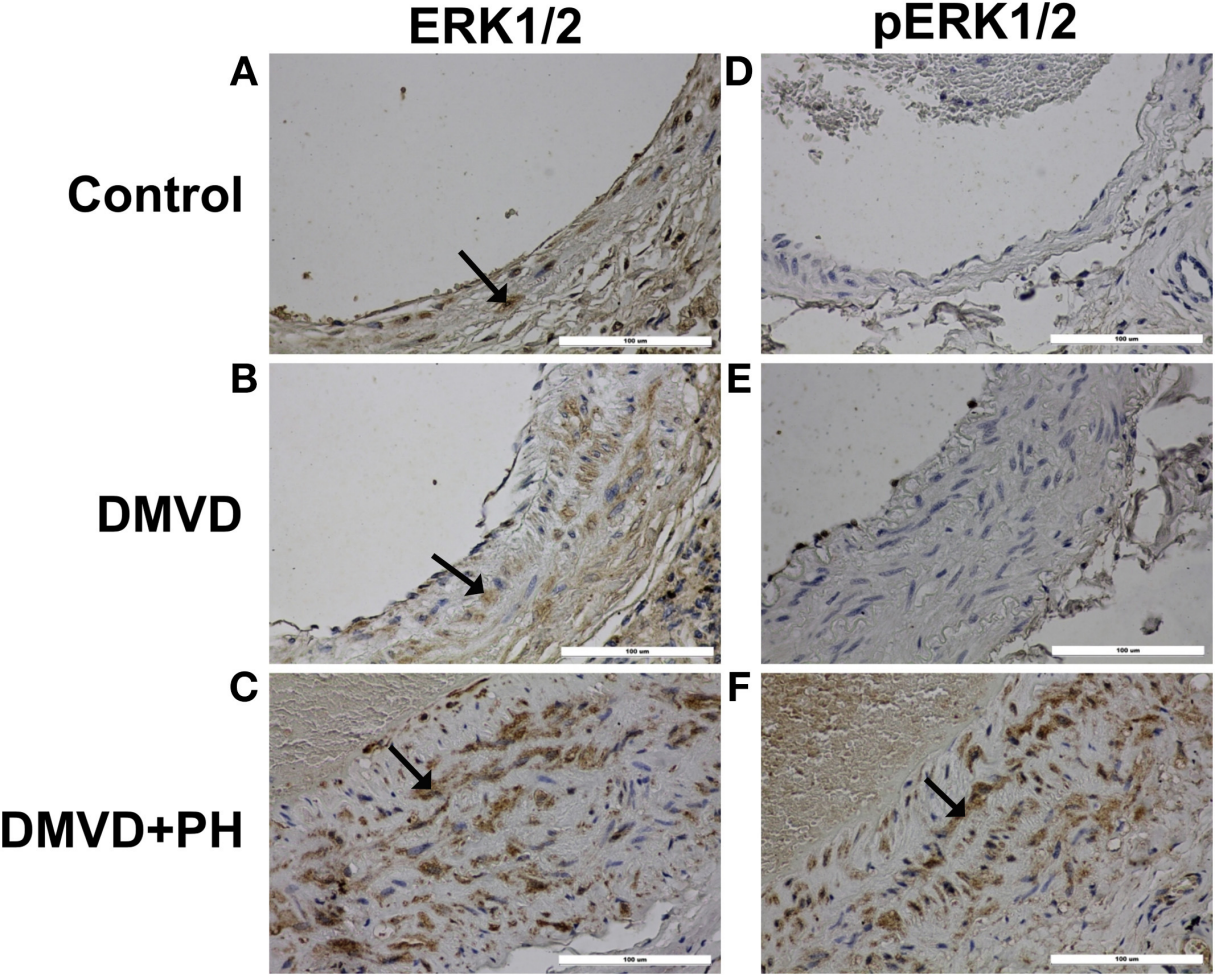
The expression of downstream molecules in 5HT pathway including extracellular regulated kinase 1/2 (ERK1/2) and phosphorylated ERK1/2 (pERK1/2) in pulmonary arterial smooth muscle cells of the control **(A,D)**, degenerative mitral valve disease (DMVD) **(B,E)** and degenerative mitral valve disease with pulmonary hypertension (DMVD+PH) groups **(C,F)**. The ERK1/2 and pERK1/2 expressed in both nucleus and cytoplasm of pulmonary arterial smooth muscle cells but predominantly located in cytoplasm presenting as brown color (Labeled streptavidin-biotin, Immunohistochemistry, Mayer's Hematoxylin counterstained, 400x magnification).

**Figure 5:**
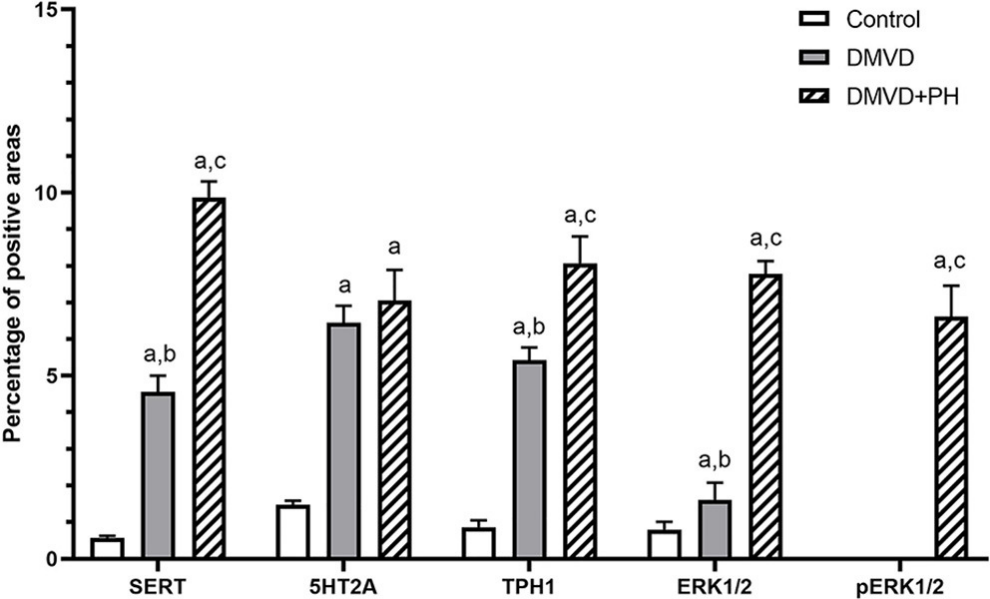
The average percentage of positive areas of molecules in 5HT pathway in the control, degenerative mitral valve disease (DMVD) and degenerative mitral valve disease with pulmonary hypertension (DMVD+PH) groups. Data are expressed as mean and standard deviation (bars). ^a^Indicate significant difference at *p* < 0.05 compared to the control group. ^b, c^Indicate significant difference at *p* < 0.05 between the DMVD and DMVD+PH groups.

The expression of SERT, 5HT2A, TPH1, ERK1/2, and pERK1/2 were found mainly in the medial layer of the pulmonary artery. The expression of SERT, 5HT2A, and TPH1 was observed in the cytoplasm of pulmonary arterial SMCs (Figure 3). ERK1/2 and pERK1/2, downstream signaling proteins of 5HT pathway were expressed in the nucleus and cytoplasm of pulmonary arterial SMCs but predominantly located in the cytoplasm (Figure 4). In the control group, the percentage of SERT, 5HT2A, TPH1, and ERK1/2 positive area was very low, and they were significantly increased in the DMVD and DMVD+PH groups (Table 2, Figure 5). Although ERK1/2 was presented in all groups, pERK1/2 was presented only in the DMVD+PH group (Figure 4). In the DMVD+PH group, the expression of SERT, TPH1, ERK, pERK was significantly increased compared to the DMVD group. Only the expression of 5HT2A that was not significantly different between the DMVD and DMVD+PH groups (Table 2, Figures 3, 5).

**Table 2:** The percentage of SERT, 5HT2A, TPH1, ERK1/2, pERK1/2 positive areas in the control, degenerative mitral valve disease (DMVD) and degenerative mitral valve disease with pulmonary hypertension (DMVD+PH) groups.

**Parameters (%)**	**Control (*n* = 5)**	**DMVD (*n* = 7)**	**DMVD+PH (*n* = 7)**	***p*-value**
SERT	0.58 ± 0.05	4.56 ± 0.44 ^a^^,^ ^b^	9.87 ± 0.43 ^a^^,^ ^c^	<0.0001
5HT2A	1.48 ± 0.11	6.45 ± 0.46 ^a^	7.06 ± 0.83 ^a^	<0.0001
TPH1	0.86 ± 0.19	5.43 ± 0.34 ^a^^,^ ^b^	8.07 ± 0.73 ^a^^,^ ^c^	<0.0001
ERK	0.80 ± 0.21	1.61 ± 0.47 ^a^^,^ ^b^	7.78 ± 0.35 ^a^^,^ ^c^	<0.0001
pERK	0.00 ± 0.00	0.00 ± 0.00 ^b^	6.61 ± 0.85 ^a^^,^ ^c^	<0.0001

*Data are expressed as mean ± standard deviation (SD)*.

The significant difference was assessed by one-way ANOVA at p < 0.05.

a *Indicate significant difference at p < 0.05 compared to the control group*.

b, c *Indicate significant difference at p < 0.05 between the DMVD and DMVD+PH groups*.

The %MT was strongly correlated with the expression of proteins related to 5HT pathway including SERT (*r* = 0.923, *p* < 0.0001), 5HT2A (*r* = 0.821, *p* < 0.0001), TPH1 (*r* = 0.932, *p* < 0.0001), ERK1/2 (*r* = 0.854, *p* < 0.0001), pERK1/2 (*r* = 0.754, *p* < 0.0001).

## Discussion

Pulmonary arterial remodeling has been reported in human patients affected with PH and PH-induced animal models (7–14, 16, 17, 33–36). Histopathological changes of the pulmonary artery affect all elements of the pulmonary arterial walls including the tunica intima, tunica media, and tunica adventitia. While intimal and adventitial remodeling was not found in all forms of PH, medial remodeling was a common pathological change found in humans affected with PH (8, 9, 11, 12, 37, 38) and PH-induced animal models (13–17). This study reveals that all dogs in the DMVD and DMVD+PH groups had increased medial thickness similar to previous studies in dogs (1, 3) and human patients affected with PH secondary to left-sided heart failure (39). Interestingly, DMVD dogs with PH had %MT greater than those without PH, suggesting further progression of medial remodeling in DMVD dogs with PH.

The serotonin pathway has been proven to relate to pulmonary vascular remodeling in humans with PH and PH-induced animal models. Serotonin signaling has been suggested as being involved with the pathogenesis of DMVD in dogs (40–44) and humans (45, 46) but very little is known about its effects on the pathogenesis of PH in dogs with DMVD. Serotonin can be used to induce PH in a canine animal model, suggesting involvement of 5HT in the pathogenesis of PH in dogs (47–52). However, there is no study of naturally occurring PH in dogs with DMVD. To the authors' knowledge, this is the first study that examining the expression of proteins related to the 5HT pathway in the pulmonary arteries of dogs affected with PH secondary to DMVD.

The results showed a strong expression of proteins related to the 5HT pathway in DMVD dogs both with and without PH compared to control dogs. The expression of SERT was predominantly located in the medial layer of the pulmonary artery. The percentage of the SERT positive area was significantly higher in the DMVD and DMVD+PH groups compared to the control group. In addition, the expression of SERT was increased in the DMVD+PH group compared with the DMVD group. Similar findings have been reported in various forms of PH in human patients including idiopathic PH (53), primary PH (18, 25) and secondary PH from various diseases (18) and in PH-induced animal models (16, 26, 54, 55). The results of the present study showed a relationship between an overexpression of SERT and the medial remodeling of the pulmonary artery in dogs affected with PH secondary to DMVD. Interestingly, DMVD dogs without PH also presented with SERT overexpression. Eddahibi et al. (38) reported that hypoxia could increase the rate of transcription of the SERT gene in pulmonary arterial SMCs. Moreover, experimental studies in mice showed that SERT was overexpressed during hypoxia (26, 56). Taken together, it is possible that hypoxia from pulmonary edema in DMVD dogs might be one of the factors that induces an increase in SERT expression in pulmonary arterial SMCs and stimulates medial remodeling.

Several studies have provided evidence that not only SERT but also 5HT receptors contribute to medial remodeling (18, 57–63). 5HT receptors can be divided into seven families (5HT1-7) (22). The 5HT2A receptor was selected for this study because it has been reported to mediate the effects of 5HT induced pulmonary arterial remodeling in animal models (61, 64). In addition, the 5HT2A receptor has been found in coronaries of dogs, and contributes to vasoconstriction induced by 5HT (65). The present study showed that 5HT2A was expressed in the medial layer of the pulmonary artery in the control, DMVD and DMVD+PH groups. The expression of 5HT2A was increased in the DMVD and DMVD+PH groups compared to the control group. In contrast to studies in human patients with PH and PH-induced mice, the expression of 5HT receptors including 5HT2A was no different between the PH and control groups (18, 59). Interestingly, the percentage of the 5HT2A positive area was no different between the DMVD and DMVD+PH groups. It might be speculated that an upregulation of 5HT2A might be constant in DMVD dogs with and without PH.

Not only 5HT2A receptor but also 5HT1B receptor has been reported to be involved with pulmonary hypertension in humans and large animal models such as pigs (20, 57, 58, 60, 62). A previous study showed that the 5HT1B receptor has a role in constriction of the coronary artery in a dog model (65). However, the relationship of 5HT1B receptor and pulmonary hypertension in dogs has not been studied yet. Further studies are needed to be performed to evaluate the expression and roles of 5HT1B in pulmonary artery of DMVD dogs with PH.

The results of this study suggest that SERT and 5HT2A may work incorporation in pulmonary arterial remodeling in DMVD dogs. Previous studies showed that SERT inhibitors reduced the proliferation of pulmonary arterial SMCs (18, 66), and they can protect the development of PH secondary to hypoxia (67) and monocrotaline induction (55). The 5HT2A antagonist reduced pulmonary arterial SMCs proliferation in both normoxic and hypoxic conditions (32). Moreover, the combination of the 5HT receptor antagonist and SERT antagonist can reduce pulmonary arterial SMCs proliferation in response to 5HT (62) and is more effective than using SERT inhibitors alone (68).

TPH1, a rate-limiting enzyme for 5HT synthesis has been suggested as being associated with PH (16). In humans, TPH1 catalyzed tryptophan to 5HT in pulmonary arterial endothelial cells (ECs) and then stimulates pulmonary arterial SMCs proliferation in a paracrine fashion causing medial remodeling (19, 20). Interestingly, the results of the present study showed that TPH1 was mainly expressed in the pulmonary arterial SMCs rather than the ECs in all three groups. On the contrary, previous studies in PH-induced animal models noted that TPH1 expression was present only in pulmonary arterial ECs but not in SMCs (32, 69). The percentage of the TPH1 positive area was very low in the control group and significantly increased in the DMVD and DMVD+PH groups. Additionally, the TPH1 positive area increased in the DMVD+PH group compared to the DMVD group. A previous study showed a weak expression of TPH1 in normoxic mice (22). Chronic hypoxia can induce an increase of TPH1 expression and synthesis (20). Taken together, it is reasonable to speculate that chronic hypoxia from pulmonary edema may be one of the factors that induce an increased expression of TPH1 in DMVD dogs.

Although the mechanisms by which SERT and 5HT receptors mediate pulmonary arterial SMCs proliferation remain unclear, several studies have suggested that 5HT transport into SMCs via SERT and 5HT receptors and intracellular accumulation of 5HT induce reactive oxygen species and Rho-kinase activating phosphorylation and nuclear translocation of ERK1/2, leading to an increase in transcription of nuclear growth factors and mediation of cellular proliferation (21, 22). Activation of ERK1/2 is an important mechanism in 5HT-induced pulmonary arterial SMCs proliferation and is associated with the pathophysiology of PH. The present study examined the expression of ERK1/2 and pERK1/2, which are downstream molecules of the 5HT pathway in the pulmonary artery. The results showed that ERK1/2 and pERK1/2 were present in the nucleus and cytoplasm of pulmonary arterial SMCs but were predominantly located in the cytoplasm. The percentage of ERK1/2 positive areas was very low in the control group but was significantly increased in the DMVD and DMVD+PH groups. However, pERK1/2 was increased only in the DMVD+PH group. The *in vivo* study of pulmonary arterial SMCs in humans (32) and rats (70) showed that ERK1/2 and pERK1/2 expression was increased in hypoxic conditions determined by western blot. An increase in ERK1/2 expression in DMVD dogs may occur secondary to chronic hypoxia from pulmonary edema. Interestingly, although, SERT, 5HT2A, TPH1, and ERK1/2 were overexpressed in DMVD dogs with and without PH, pERK1/2 was increased only in DMVD dogs with PH. These results suggest that activation of the 5HT signaling pathway through ERK1/2 may occur mainly in DMVD dogs with PH.

Several studies in culture cells demonstrated that TPH1 (53) and SERT inhibitors could reduce pulmonary arterial SMCs proliferation (18, 66). Moreover, an inhibition of TPH1 and SERT can reduce the development of PH secondary to hypoxia and monocrotaline induction in animal models (55, 67). The present study reported the association between PH in DMVD dogs and an upregulation of proteins related to 5HT pathway. The selective inhibitors of TPH1, SERT and 5HT2A receptor may be the target of interests for further investigation to reduce the development of PH secondary to DMVD.

In conclusion, medial remodeling of the pulmonary artery in DMVD dogs with PH is associated with an upregulation of proteins related to the 5HT signaling pathway. Further studies are necessary to investigate the mechanisms of the 5HT signaling pathway in mediating PH in dogs affected with DMVD. A better understanding of the pathogenesis of PH in DMVD dogs will provide important insight into its management such as development target therapy or selective inhibitors for treatment or prevent PH secondary to DMVD in the future.

A limitation of this study was that the immunohistochemical study used an antibody against other species including humans, rabbits and rats instead of dogs. As because there are no available commercial antibodies against dogs and based on the amino acid sequence provided by UniProt, canine SERT, 5HT2A, TPH1, ERK1/2, and pERK1/2 are highly homologous (>90%) to other species. Moreover, these antibodies have been successfully used in DMVD dogs (27, 28, 71). Therefore, this antibody is reasonable for use in this present study.

## Data Availability Statement

The original contributions presented in the study are included in the article/supplementary materials, further inquiries can be directed to the corresponding author.

## Author Contributions

All authors listed have made a substantial, direct and intellectual contribution to the work, and approved it for publication.

## Conflict of Interest

The authors declare that the research was conducted in the absence of any commercial or financial relationships that could be construed as a potential conflict of interest.

## Abbreviations

DMVD: degenerative mitral valve disease
ECs: endothelial cells
ERK 1/2: extracellular regulated kinase 1/2
5HT, 5-hydroxytryptamine: serotonin
5HT2A: Serotonin 2A receptor
%MT: percentage of medial thickness
PAP: pulmonary arterial pressure
pERK 1/2: phosphorylated extracellular regulated kinase 1/2
PH: pulmonary hypertension
SD: standard deviation
SERT: serotonin transporter
SMCs: smooth muscle cells
TPH1: tryptophan hydroxylase 1.
