# Corrigendum: The Expression of Proteins Related to Serotonin Pathway in Pulmonary Arteries of Dogs Affected With Pulmonary Hypertension Secondary to Degenerative Mitral Valve Disease

**DOI:** 10.3389/fvets.2021.655751

**Published:** 2021-02-09

**Authors:** Siriwan Sakarin, Sirilak Disatian Surachetpong, Anudep Rungsipipat

**Affiliations:** ^1^Department of Veterinary Medicine, Faculty of Veterinary Science, Chulalongkorn University, Bangkok, Thailand; ^2^Companion Animal Cancer Research Unit, Department of Veterinary Pathology, Faculty of Veterinary Science, Chulalongkorn University, Bangkok, Thailand

**Keywords:** degenerative mitral valve disease, hyperplasia, pulmonary artery, pulmonary hypertension, serotonin, smooth muscle cells

In the original article, we neglected to include the funder **The Thailand Research Fund**, **RSA6080009** to author **Sirilak Disatian Surachetpong**.

The authors apologize for this error and state that this does not change the scientific conclusions of the article in any way. The original article has been updated.

